# Mitochondrial DNA Indicates Late Pleistocene Divergence of Populations of *Heteronympha merope*, an Emerging Model in Environmental Change Biology

**DOI:** 10.1371/journal.pone.0007950

**Published:** 2009-11-24

**Authors:** Melanie Norgate, Jay Chamings, Alexandra Pavlova, James K. Bull, Neil D. Murray, Paul Sunnucks

**Affiliations:** 1 School of Biological Sciences and Australian Centre for Biodiversity, Monash University, Clayton, Victoria, Australia; 2 Department of Genetics, La Trobe University, Bundoora, Victoria, Australia; University of Dayton, United States of America

## Abstract

Knowledge of historical changes in species range distribution provides context for investigating adaptive potential and dispersal ability. This is valuable for predicting the potential impact of environmental change on species of interest. Butterflies are one of the most important taxa for studying such impacts, and *Heteronympha merope* has the potential to provide a particularly valuable model, in part due to the existence of historical data on morphological traits and glycolytic enzyme variation. This study investigates the population genetic structure and phylogeography of *H. merope*, comparing the relative resolution achieved through partial DNA sequences of two mitochondrial loci, COI and ND5. These data are used to define the relationship between subspecies, showing that the subspecies are reciprocally monophyletic. On this basis, the Western Australian subspecies *H. m. duboulayi* is genetically distinct from the two eastern subspecies. Throughout the eastern part of the range, levels of migration and the timing of key population splits of potential relevance to climatic adaptation are estimated and indicate Late Pleistocene divergence both of the Tasmanian subspecies and of an isolated northern population from the eastern mainland subspecies *H. m. merope*. This information is then used to revisit historical data and provides support for the importance of clinal variation in wing characters, as well as evidence for selective pressure acting on allozyme loci phosphoglucose isomerase and phosphoglucomutase in *H. merope*. The study has thus confirmed the value of *H. merope* as a model organism for measuring responses to environmental change, offering the opportunity to focus on isolated populations, as well as a latitudinal gradient, and to use historical changes to test the accuracy of predictions for the future.

## Introduction

Butterflies are one of the most important taxa for studying the effects on species of environmental change due to their sensitivity to temperature and their history of extensive population monitoring [1]. The study of butterflies has provided convincing evidence of the impact of forces such as climate change on range distribution and extinction risk [2], [3]. Since many butterflies are relatively convenient research organisms, they have also been used for several key studies that identify proximal factors to which organisms respond under environmental change [4], [5].

**Figure 1:**
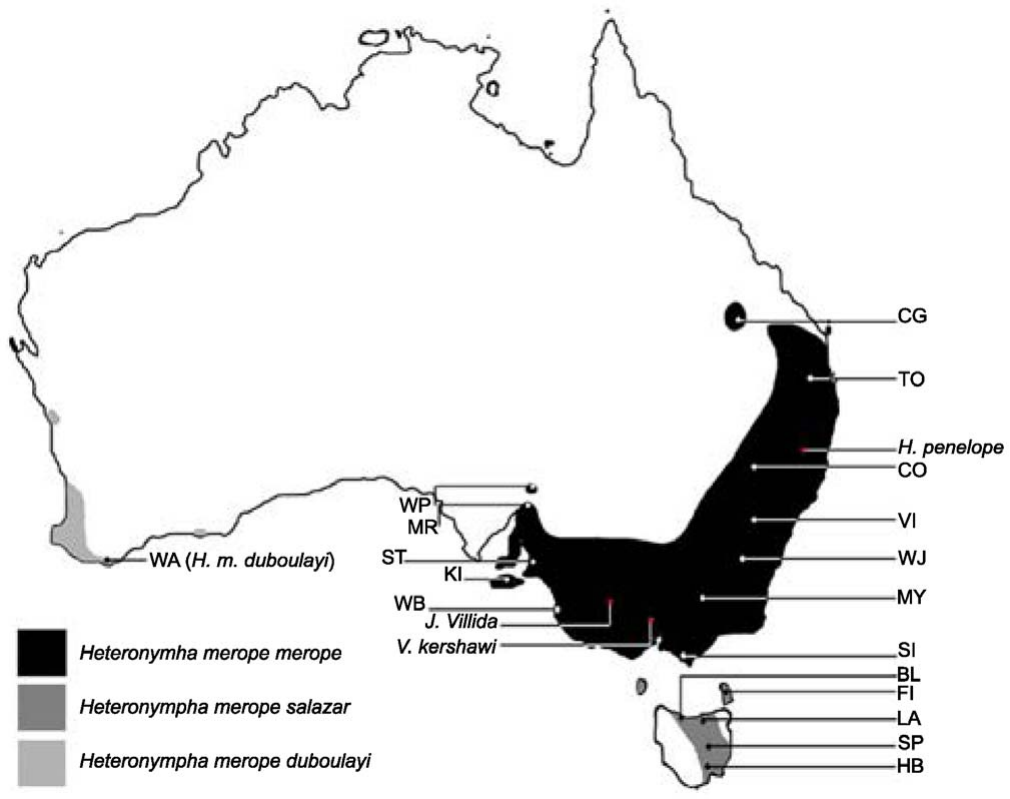
*Heteronympha merope* distribution and collection site locations. The ranges of the three subspecies are marked as shown in the key with ranges adapted from Braby [8] and Pearse [7] to show the geographic isolation of WP and CG. Sites are labelled according to abbreviated location names for *H. merope* and species names for outgroups. In the text, “eastern” refers to *H. m. merope* and *H. m. salazar* and “South Australian” refers to the State of South Australia which includes WP, MR, ST, and WB. Further details for all sites, including full location and species names, are provided in Table 1.

The Australian endemic butterfly, *Heteronympha merope*, is an exceptional study organism for this purpose because research in the late 1970s provided historical records of wing morphology and allozyme allele variation over the entire species range from field sites located near weather stations [6], [7]. *Heteronympha merope* is common and widespread over 20 degrees latitude (Figure 1) and has several congenerics of limited habitat tolerance, including some of conservation concern [8]. Isolated populations at the extremes of the wide latitudinal distribution and three geographically isolated subspecies (Figure 1) provide useful comparisons within the species. *Heteronympha merope* thus offers the potential to investigate adaptive responses to environmental change across an environmental gradient as well as in the context of barriers to dispersal.

Investigation of *H. merope* wing morphology over its entire range in the late 1970s identified a north-south cline associated with winter humidity and an east-west cline associated with yearly rainfall [7]. Indirect evidence suggests that these patterns are the result of natural selection rather than stochastic processes (e.g. the combination of characters contributing to each cline are independently inherited), but it remains possible that factors such as isolation by distance contribute substantially to the clinal variation. Knowledge of the population genetic structure would resolve this issue by providing information on gene flow throughout the range as a point of comparison with traits that may be under selective pressure.

The only available information on *H. merope* population genetic structure comes from three allozymes [6]. These allozymes display fairly uniform frequencies throughout the contiguous distribution of the eastern mainland subspecies *H. m. merope* and the Tasmanian subspecies *H. m. salazar*, but differ significantly in two geographically isolated populations of *H. m. merope* and in the Western Australian subspecies *H. m. duboulayi*. Morphological and allozymic variation are consistent in suggesting divergence of *H. m. duboulayi* from the eastern subspecies, but are otherwise uncorrelated. The lack of structure in allozyme data throughout most of the species range thus supports the hypothesis of an association with climatic variation as the underlying cause of the clines observed in wing characters rather than isolation by distance. However, two of the allozymes examined in *H. merope*, phosphoglucose isomerase (PGI) and phosphoglucomutase (PGM), have subsequently been shown to be important in thermal biology and flight activity in butterflies [reviewed by 9]. This has two implications. First, as the allozymes are likely to be under direct selective pressure from climate change, other genetic markers must be investigated to gain a more accurate picture of historical demographic processes in these butterflies. Second, this species is an ideal candidate for examining these adaptive loci as well as morphological characters over 30 years of climate change in the context of a backdrop provided by neutral genetic markers. Spatial genetic and phylogeographic analysis within the species is required to support this approach.

Despite caveats about the application of mitochondrial DNA (mtDNA) in population genetics and phylogenetics [10], [11], it is in common use owing to strengths including relative ease of amplification from old specimens, considerable reference information, and relatively fast sorting among lineages. The NADH dehydrogenase group of mtDNA genes (ND1-6) and the Cytochrome c oxidase group (CO1-3) have been used extensively for phylogenetics and population genetics, including within closely-related Lepidoptera [12]–[16]. Some studies have also suggested particular suitability of ND5 for studies of Lepidoptera at low taxonomic levels [17], [18].

This study investigates the population genetic structure and phylogeography of *H. merope* using mtDNA applied to samples over the continent-wide range of the species, with comparison to congeners. The results provide an understanding of the history of subspecies divergence and the isolation of populations at the extremes of the range distribution. This knowledge of the population genetic structure addresses questions about the adaptive significance of clines in morphological characters in *H. merope* as well as providing valuable baseline data for the further study of thermal biology and dispersal potential in butterflies.

## Methods

### Ethics statement

Butterflies were collected under permits issued by The Victorian Department of Sustainability and Environment (10004062), New South Wales Department of Environment and Climate Change (S12221), Forestry New South Wales (CO34155), South Australian Department for Environment and Heritage (K25349 2) and Queensland Parks and Wildlife Service (WITK04310307), and adheres to Monash University and Australian legal requirements for research on Lepidoptera that are not of conservation concern.

### Specimen collection

Adult *H. merope* were collected from 18 sites representing the entire species range, 10 of which are the same or near to sites sampled in previous studies of this species [6], [7], [19]. The congeneric *H. penelope* and representatives of two other Nymphalidae, *Vanessa kershawi* and *Junonia villida*, were opportunistically collected to provide outgroups. Sampling locations and collection details are shown in Figure 1 and Table 1. Most specimens were transported live and stored at −80°C. The treatment of ∼20 specimens with acetone had no effect on DNA quality. Butterflies from Flinders Island were collected in 1988 and are likely to have been killed in ethyl acetate, then stored in the presence of naphthalene (provided by Tim New, La Trobe University).

**Table 1:** Collection locations and numbers for butterfly specimens used in this study.

Collection Site		Latitude	Longitude	COI	ND5	Both loci
CG	Carnavon Gorge	25° 3′ 35.41″	148° 14′ 1.71″	15	19	15
CO	Coonabarabran	31° 15′ 15.99″	149° 17′ 4.78″	-	17	-
FI	Flinders Island	39°53′34.36″	148° 3′ 6.33″	-	17	-
KI	Kangaroo Island	35° 47′ 19.87″	137° 12′ 0.04″	-	30	-
MR	Mt Remarkable	32° 55′ 28.39″	138° 5′ 33.54″	-	26	-
MY	Myrtleford	36° 33′ 48.73″	146° 44′ 26.39″	10	22	10
SI	Shallow Inlet	38° 51′ 0.45″	146° 11′ 17.64″	26	28	24
ST	Stirling	35° 1′ 3.56″	138° 42′ 42.17″	22	23	18
TO	Toowoomba	27° 34′ 52.80″	151° 59′ 19.40″	16	23	14
VI	Vittoria	33° 25′ 34.96″	149° 18′ 50.76″	10	9	9
WJ	Wee Jasper	35° 6′ 54.00″	148° 40′ 4.80″	-	10	-
WP	Wilpena Pound	31° 29′ 39.00″	138° 38′ 40.00″	27	43	27
WB	Wrights Bay	37° 1′ 40.43″	139° 47′ 58.52″	9	21	8
BL (*H. m. salazar*)	Black River	40° 50′ 59.70″	145° 18′ 32.23″	-	19	-
HB (*H. m. salazar*)	Hobart	42° 55′ 51.46″	147° 21′ 24.08″	11	24	9
LA (*H. m. salazar*)	Launceston	41° 27′ 17.37	147° 10′ 0.59″	10	40	-
SP (*H. m. salazar*)	St Peter's Pass	42° 16′ 9.00″	147° 24′ 21.00″	10	11	10
WA (*H. m. duboulayi*)	Albany, WA	35° 6′ 6.96″	117° 54′ 18.98″	4	5	4
*H. penelope*	Armidale, NSW	30° 34′ 52.24″	151° 44′ 35.39″	2	5	2
*Vanessa kershawi*	Daylesford, Vic	37° 20′ 38.25″	144° 12′ 23.56″	-	1	-
*Junonia villida*	Horsham, Vic	36° 43′ 16.58″	142° 20′ 19.92″	-	1	-

Collections are *H. m. merope* unless otherwise marked. All specimens except FI were collected between November 2007 and January 2008. FI specimens were collected in 1988. For *H. merope* 108 females and 58 males were screened COI, and 253 females and 134 males were screened for ND5.

### DNA extraction

For all except the Flinders Island specimens, DNA was isolated using a modified salting out protocol [20]. Two legs per specimen were crushed to damage the exoskeleton and DNA was extracted as described previously [21]. The only alterations were that initial incubation was in 600 µl of TNES buffer and that an additional incubation at room temperature for 15 min after the addition of 5M NaCl was necessary to ensure no further precipitation following the next step. DNA was resuspended in 400µl of Tris EDTA buffer (TE).

DNA was isolated from one leg of each Flinders Island specimen using a DNeasy Tissue kit (Qiagen), according to the manufacture's instructions for insect tissue, with minor alterations. To maximise DNA yield given the small amount of tissue and the age of the specimens, samples were initially ground in 50 µl of buffer and incubated at 55°C for 48–60 h, and the final elution volume was 20 µl TE. The average yield was 24.3 ng/µl, with an additional 6.5 ng/µl recovered in a second 30µl elution (measured using a NanoDrop 1000, Thermo Scientific).

### Screening for sequence variation in COI and ND5

A 470 bp COI region and a 450 bp ND5 region were PCR amplified from 2 µl of DNA in a 10 µl final volume for Single Stranded Conformation Polymorphism (SSCP) analysis, or from 5 µl of DNA in a 25 µl final volume for direct sequencing. The PCR primers have been published previously: COI primers were ‘mtD6’ C1-J-1718 and ‘mtD9’ C1-N-2191 [22]; ND5 primers were ‘V1’ and ‘C2’ [23]. For all except the Flinders Islands specimens, PCR reactions contained 0.5 µM of each primer, 2 mM MgCl_2_, 0.2 mM dNTPs, 0.4 mg/ml BSA (Invitrogen), 0.25 units *Taq* polymerase (Fermentas), 10 mM Tris-HCl and 50 mM KCl and 0.08% (v/v) Nonidet P40. Both loci were amplified for 30 cycles with a 50°C primer annealing temperature and a 30 s polymerase extension time.

When using SSCP analysis to assess PCR products for sequence variation, 0.05 µl of ^33^P-labelled alpha-dATP (Perkin Elmer) was also included, following a previously described protocol [24]. Samples were run for 16–18 h at 10 W. Representative samples of each haplotype were commercially sequenced using either the C1-J-1718 primer for COI or the V1 primer for ND5 (Macrogen Inc, Seoul, Korea), with sufficient replication of common haplotypes to ensure the SSCP screen had reliably detected single nucleotide substitutions. In all cases, SSCP identified and distinguished all haplotypes found through direct sequencing.

Successful amplification of ND5 from each Flinders Island specimen was achieved using 15 µl of DNA in a 50 µl reaction containing 0.5 µM each primer, 0.2 mM dNTPs, 0.2 mg/mL BSA, 1 unit Velocity DNA polymerase (Bioline), and the Velocity HiFi reaction buffer (Bioline), which has a final concentration of 2 mM MgCl_2_. Cycling conditions were: 97°C for 1 min; 30 cycles of 97°C for 30 s, 54°C for 30 s and 72°C for 8 s; then 72°C for 5 min. To remove PCR artefacts generated from the DNA template, the expected 450 bp product was excised from a 1% agarose gel and recovered into 100 µl TE by incubation at 37°C for 30 min. Five µl of the recovered PCR product was then re-amplified for commercial sequencing with a 25 µl final volume.

### DNA sequence analysis

All sequences were edited with reference to chromatograms and aligned in MEGA v4 [25] using the ClustalW algorithm [26]. COI sequences were edited to 435 bp and ND5 sequences to 395 bp. To verify the identity of the amplified regions, the National Center for Biotechnology Information (NCBI) nucleotide collection (nr/nt) database was queried with the sequence of the most common haplotype using a BlastN search with default settings (NCBI, http://www.ncbi.nlm.nih.gov). This confirmed the identity of the 435 bp mtD6-mtD9 COI region. Of the 100 most similar sequences in the NCBI nucleotide sequences database, the first two encode *H. merope* COI (Genbank accession numbers AY218243 and EU920736, 100% identity) and the remainder are other Nymphalidae COI sequences (98–100% identity; Expectation Values, 5×10^−158^ to 2×10^−175^). No significant similarity to other loci is found. The 395 bp ND5 region sequenced from *H. merope* aligns well with other Lepidopteran mitochondrial ND5 sequences. The 100 most similar sequences encode Lepidopteran ND5 (87–89% identity; Expectation Values, 2×10^−132^ to 1×10^−145^), with seven of eight genera sharing the Nymphalidae family with *Heteronympha*. The three most similar species are *Lethe diana* (AB107980), *Erebia callias* (EU037820), and *Erebia niphonica* (AB303900). No significant similarity to other loci is found.

DnaSP v4.50.3 [27] was used to calculate the number of variable sites, nucleotide diversity (π), average number of nucleotide differences (k), proportion of variable sites, and proportion of synonymous and non-synonymous replacements. MEGA v4 [25] was used to calculate average nucleotide composition, pairwise differences between haplotypes and transition/transversion bias.

The *H. merope* ND5 sequences were examined for neutral protein evolution using the McDonald and Kreitman test [28] in DnaSP. Demographic processes were then investigated for each collection site using Tajima's *D* [29] and Fu's *F* [30] in Arlequin v3.1 [31] with 10,000 simulated samples. Arlequin v3.1 was also used for Mismatch analysis [32] to test the null hypothesis of demographic expansion on sites with a sample larger than 10 specimens, which excludes only Vittoria (VI; Table 1).

Partitioning of variance within and among populations was estimated using Arlequin v3.1 by analysis of molecular variance (AMOVA) with 10,000 permutations. Pairwise differentiation between sites was calculated using both *F*_ST_ and Φ_ST_. *F*_ST_ is calculated using haplotype frequency, whereas Φ_ST_ is calculated using both frequency and the level of sequence variation [33]. The comparison between the two is a useful approach for inferring relative temporal information [e.g. 34]. Relationships between the observed haplotypes were assessed by constructing Median-Joining networks. Roehl haplotype data files (RDF) were created with DnaSP and imported into Network v4.5.0.1 (Fluxus-Technology, www.fluxus-engineering.com) and networks were calculated with the Median-Joining algorithm using maximum parsimony post-processing [35].

### Estimation of time since population divergence, effective sizes, and gene flow

To examine gene flow throughout the *H. m. merope* and *H. m. salazar* distribution, three separate analyses were performed: (1) the split between mainland (n = 135) and Tasmania (n = 31) was analysed with COI data; (2) the same split was also analysed with ND5 data on a larger sample of individuals (n = 241 mainland, n = 94 Tasmania); and (3) the split between northern mesic outlier Carnarvon Gorge (n = 19) and remaining mainland individuals (n = 222) was analysed using ND5 data.

The computer program IMa [36] was used to fit an isolation-with-migration model, which assumes that an ancestral population of size θ_A_ split into two populations of sizes θ_1_ and θ_2_, t generations ago, and that after divergence the two populations receive immigrant genes from each other with rates of *m*1 and *m*2. The six demographic parameters estimated by IMa are scaled by mutation rate μ: θ = 4N_e_μ for nuclear genes, where N_e_ is effective population size; *t* = tμ, where t is number of generations since population divergence; *m* = m/μ, where m is number of immigrants per gene per generation. To convert the estimates of IMa into demographic units, an inheritance scalar of 0.25 for mitochondrial data (i.e. θ = N_e_μ) and generation time of one year were used; mutation rate μ_COI_ for COI was assumed to be 6.504×10^−6^ substitutions per year per locus, which is equivalent to the average invertebrate COI rate of 1.5% substitutions per site per lineage per million years [37]. The mutation rate for the faster-evolving ND5 gene (9.459×10^−6^ per year for the locus, or 2.395% substitutions per site per lineage per MY) was estimated from the COI rate using equation μ_ND5_ = (θ_COI_×μ_COI_)/θ_COI_, and population parameter θ (Theta Waterson, estimated from number of segregating sites θ_COI_ = 0.0045, θ_ND5_ = 0.0072) was calculated in DNAsp from COI and ND5 sequences for the group of 151 individuals from mainland and Tasmania for which both loci had been sequenced. Several preliminary metropolis-coupled Monte Carlo Markov chain simulations were required to optimize maximum parameter limits. For each of the three analyses, four replicate runs were performed (in each, MCMC simulations were run employing ten chains for 5mln steps after a 1mln steps of initial burn-in, parameters were recorded each 500^th^ step) and their marginal parameter distributions were compared for convergence. For each analysis, the final L-mode run estimated joint marginal and multi-dimensional posterior probabilities from 40,000 resulting trees. ‘Nested models’ [36] were also examined and compared to the full model using likelihood ratio test (LRT) to identify the simplest model that explains the data.

Reliability of the six parameters was assessed by examining the four replicate runs for convergence as a peak in the marginal distribution. The tail of the distribution reaching zero indicates that the most likely value does not lie outside the range examined. A more detailed description of the interpretation of IMa analysis has been published previously [36].

## Results

### Relative variation at two mitochondrial loci in *H. merope*

#### *H. merope* has limited genetic diversity at the COI region assessed

The 435 bp mtD6-mtD9 COI region was screened for 172 specimens, comprising 135 *H. m. merope*, 31 *H. m. salazar*, four *H. m. duboulayi* and two of the congeneric *H. penelope*. A total of 31 variable sites were found across the three *H. merope* subspecies, all single nucleotide substitutions. None of the substitutions translate into stop codons, there is a strong transition bias (6.62; 27 transitions and four transversions), and the majority are synonymous. Three non-synonymous substitutions were detected (Table S1), but all at sites that appear to be under minimal functional constraint [38]. The sequenced region therefore appears to be derived from *H. merope* mitochondrial COI and not from a nuclear pseudogene [21].

Eleven of the nucleotide substitutions contribute to 13 haplotypes across the *H. m. merope* and *H. m. salazar* specimens (Table S1), seven of which were singletons (found in only one individual each in the sample). Any two of these haplotypes differ by an average of 0.395 nucleotide substitutions (Table 2). In contrast, the specimens from the Western Australian subspecies, *H. m. duboulayi*, share a single haplotype that differs from the most common *H. m. merope* and *H. m. salazar* haplotype at 25 sites. This indicates that *H. m. duboulayi* is considerably divergent from the other two subspecies, as is the congeneric *H. penelope* which differs from the most common *H. m. merope* and *H. m. salazar* haplotype at 34–35 sites. Only the most common haplotype (present in 80.7% of specimens) was shared between *H. m. merope* and *H. m. salazar*. The low frequency of each of the other 12 haplotypes (0.6–7.8%), limits the potential for detailed analysis of the genetic structure of these subspecies using mtD6-mtD9 COI.

**Table 2:** Genetic distances between outgroup haplotypes and most common *H. m. merope*/*salazar* haplotype.

	COI		ND5	
Species	Haplotype	Difference from COI H1 (bp)	Haplotype	Difference from ND5 H1 (bp)
*H. m. merope* and *H. m. salazar*	H1-H31 (n = 166)	0.395* (0.1%)	H1-H13 (n = 382)	2.84* (0.7%)
*H. m. duboulayi*	WA1 (n = 4 )	25 (5.8%)	WA (n = 4)	21 (5.3%)
	-	-	WA2 (n = 1)	20 (5.1%)
*H. penelope*	Hpenelope1 (n = 1 )	34 (7.8%)	Hpenelope1 (n = 3 )	28 (7.1%)
	Hpenelope2 (n = 1 )	35 (8.1%)	Hpenelope2 (n = 2 )	28 (7.1%)
*Junonia villida*	-	-	Jvillida (n = 1)	49 (12.4%)
*Vanessa kershawi*	-	-	Vkershawi (n = 1)	50 (12.7%)

* Values for H1-H31 are average difference between any two *H. m. merope* or *H. m. salazar* haplotypes, including H1; all other values are the difference from H1, which is the most common haplotype and shared by *H. m. merope* and *H. m. salazar*. n = number of specimens from Table 1 with a given haplotype. The percentage divergence is shown in brackets for each.

#### ND5 provides a suitable genetic marker for *H. merope*

The 395 bp ND5 region was screened in 375 specimens, comprising 288 *H. m. merope*, 75 *H. m. salazar*, five *H. m. duboulayi*, five *H. penelope*, and one specimen each of Nymphalidae *Vanessa kershawi* and *Junonia villida*. A total of 39 single nucleotide substitutions were found within the three *H. merope* subspecies, 25 of these within *H. m. merope* and *H. m. salazar* (Table S2). None translate into stop codons, there is a strong transition bias (6.01; 37 transitions and four transversions), and only three non-synonymous substitutions were detected. Thus the sequenced region appears to be derived from *H. merope* mtDNA [21].

*Heteronympha m. merope* and *H. m. salazar* together contribute 31 haplotypes which, with the exception of 19 haplotypes detected in single individuals, are all present in at least one individual from each of the two subspecies. The average difference between any two haplotypes is 2.84 nucleotide substitutions (Table 2). *H. m. duboulayi* differs from the most common of these at 20–21 sites and *H. penelope* differs at 28 sites. In comparison, two other Nymphalidae, *Vanessa kershawi* and *Junonia villida*, differ by 49–50 nucleotide substitutions. Consistent with data from COI, the Western Australian *H. m. duboulayi* ND5 sequence therefore appears almost as divergent from the other two subspecies as is that of the congeneric *H. penelope*.

The ND5 locus was more highly resolving than COI as it showed more sequence variation: nucleotide diversity (π) and average number of nucleotide differences (k) were 3.2 and 2.9 times higher for ND5, respectively (Table 3). No significant departure from neutrality was detected for ND5 by McDonald and Kreitman test when *H. m. merope* and *H. m. salazar* were compared to either *H. penelope* or *H. m. duboulayi*, or when *H. m. duboulayi* was compared to *H. m. penelope* (Fisher's exact test, P>0.1 for each). However, neutrality tests Fu's *F* or Tajima's *D* [39] were significant for Coonabarabran (Fu's *F* = −1.936, P = 0.012), Stirling (Fu's *F* = −2.696, P = 0.028), Vittoria (Tajima's *D* = −1.610, P = 0.036) and Kangaroo Island (Tajima's *D* = −1.147, P = 0.037), which can indicate either purifying selection or population expansion. Mismatch analysis indicates that the model of population expansion could not be rejected for any site (P>0.05) except Toowoomba (P<0.001). As Toowoomba did not yield a significant Fu's *F* or Tajima's *D* value, there is no evidence for departure from neutrality at this locus.

**Table 3:** Comparison of 435bp region of COI and 395bp region of ND5.

	N	L	M	k	V_s_ k	k/L*	k/N*	π	SD of π	π/L†
COI (mtD6)	166	435	11	0.395	0.002	0.0009	0.0024	0.00091	0.00014	2.09×10^−06^
ND5 (V1)	198	395	18	1.138	0.006	0.0029	0.0057	0.00288	0.00023	7.29×10^−06^

Statistics are based on average differences between any two randomly selected *H. m. merope* and *H. m. salazar* haplotype sequences. N, number of sequences; L, length of sequence; M, number of variable sites; k, average number of nucleotide differences; V_s_ k sampling variance of k; k/L k over length of sequence*; π nucleotide diversity; SD π standard deviation of π.

* The value of k is dependent on L and N so k/L and k/N provide values standardised by sequence length and the number of sequences, respectively [63].

† The value of π is dependent on L, thus π/L may be a more accurate comparison [63].

Thus the ND5 region examined here provides a suitable mtDNA marker in this species, with more resolution than COI. The following results are derived from ND5 alone for all analyses except IMa, where COI was included to provide additional information on mutation rates (Methods). Similar analyses performed with COI and with the combined ND5 and COI sequences where both were available provide consistent data, although the COI data has lower resolution as expected (Figure S1). The ‘Barcoding of Life’ region of COI, which spans the region described above, was also investigated but found to have less resolution than ND5 so it was not analysed in detail. A list of all sequenced *Heteronympha* specimens and the corresponding haplotypes is provided in Table S3. As most of the sequence diversity in *H. merope* occurs between western and eastern subspecies, *H. m. duboulayi* has been treated as an outgroup for further analysis of the population genetic structure throughout the eastern range of the species.

### ND5 phylogeography in *H. m. merope* and *H. m. salazar*

#### The evolutionary relationships among haplotypes and *H. merope* population structure

**Figure 2:**
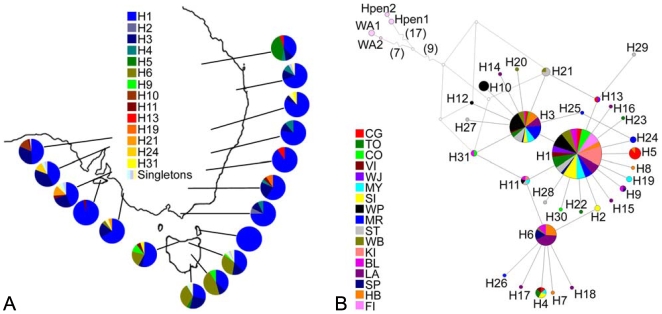
Relationships among haplotypes and collection sites. (A) Distribution of ND5 haplotypes amongst collection sites. Haplotypes are labelled by colour and the frequency at each site is indicated in pie charts. H1 (bright blue) is present at all sites and H7-H8, H12-H18, H20, H22-H23, and H25-H30 (represented in three pastel colours for simplicity) are present in a single individual each. (B) Median Joining network indicating the relationship amongst ND5 haplotypes. Each pie chart represents a different haplotype, made up of collection sites in which that haplotype occurs (sites labelled by colour). Haplotypes connected by a line differ in sequence by one base pair. The size of each pie chart indicates the relative frequency of the haplotype within the entire sample. The size of the collection site slices is influenced by both the frequency of the haplotype across the sites and the sample size for each site. As sample sizes are not equal (Table 1), slices may be interpreted as indication of presence of a haplotype at a site rather than frequency. The network shown is estimated as being connected to outgroups *H. m. duboulayi* (WA1 and WA2) and *H. penelope* (Hpen1 and Hpen2) via inferred common ancestors of H12, H21, and H31 (open circles).

Differences in sequence and frequency can be used to investigate the evolutionary relationships among haplotypes. Only the most common haplotype, H1 (Genbank accession number GQ922133), is found in all sampling sites and accounts for 59% of specimens (Figure 2A). Collection sites where H1 account for fewer than 50% of haplotypes include Tasmanian sites (*H. m. salazar*); Stirling (ST), Mount Remarkable (MR) and Wilpena Pound (WP) in the State of South Australia; and the geographically isolated site at the northern extreme of the range, Carnarvon Gorge (CG). Haplotype H6 is most common in all sites on the large island of Tasmania, and is seen otherwise only in a single mainland individual from Wright Bay (WB) in South Australia.

By incorporating differences in sequence as well as frequency, haplotype relationships can be graphically depicted in a Median Joining network (Figure 2B). The network comprises three interconnected star-shaped phylogenies centring on H1, H3 (Genbank accession number GQ922135) and H6 (GQ922138). H3 is the second most common haplotype, present in 17% of individuals and 81% of sites. The star-shaped phylogenies suggest that most haplotypes are related directly to H1, H3 or H6. Although there is much similarity between sites, and *H. m. salazar* clearly shares haplotypes with *H. m. merope*, the distribution of haplotypes in the network diagram shows some association with geographic population structure. Haplotypes present either exclusively or in high frequency in sites from mainland South Australia are clustered together in the network diagram (haplotypes: H3, H10-12, H20-21, H24-29; sites: WP, MR, ST, WB). Most of these South Australian haplotypes are related to H3, which is more frequent in these sites relative to most of the eastern sites (Figure 2A). Tasmanian haplotypes are related to either H6 or H1. The ancestry of H6 is unclear, as the sequence relationships show that either H2 (GQ922134) or H11 (GQ922143) could provide intermediate haplotypes linking H6 to H1.

Differences in haplotype sequence and frequency were further investigated to estimate the degree of gene flow within the population as a whole (Table 4). Most variation occurs within rather than among sites, implying low genetic structure in the sampled set. However, significant *F*_ST_ (frequency-based) and Φ_ST_ (frequency- and sequence-based) measures of variation are indicative of some restriction in historical as well as contemporary gene flow (P<0.0001 for both *F*_ST_ and Φ_ST_). A hierarchical AMOVA with Tasmanian sites and mainland sites divided into two groups showed significant structuring between the groups in addition to among all sites (Table 4). Some of the variation within the *H. m. merope/H. m. salazar* range (Table 4) can therefore be explained by differences between Tasmania and the mainland. Compared to the *F*_ST_-based result, Φ_ST_ indicates that a larger portion of within-site variation is explained by the grouping of Tasmanian and mainland sites, supporting the hypothesis of genetic separation between regions on the timescale over which ND5 evolves new sequences.

**Table 4:** Molecular variance in *H. m. merope* and *H. m. salazar*.

*H. m. merope* and *H. m. salazar* pooled				
	Within sites (%)	Among sites (%)	F_ST_/ΦST	P-value
*F*_ST_	86.74	13.26	0.13264	<0.0001
Φ_ST_	85.28	14.72	0.14717	<0.0001

*H. m. merope* and *H. m. salazar* grouped separately									
	Within sites (%)	Within groups (%)	Among groups (%)	*F*_ST_/Φ_ST_ *	P-value	*F*_SC_/Φ_SC_ *	P-value	*F*_CT_/Φ_CT_ *	P-value
*F*_ST_	80.72	6.48	12.8	0.19283	<0.0001	0.07431	<0.0001	0.12803	<0.001
Φ_ST_	76.03	6.24	17.73	0.23966	<0.0001	0.07582	<0.0001	0.17728	<0.01

Flinders Island has been excluded from this analysis as relationship to the Tasmanian locations is unclear. A separate AMOVA performed with Flinders Island included verified that the overall *F*_ST_/Φ_ST_ would not be significantly affected. Groups used separately are (1) all mainland sites including Kangaroo Island, and (2) all Tasmanian sites. Within site variation is not identical between the two analyses despite the statistic being the same, as some of this variation is now explained by the differences between groups.

* T, total population; S, sub-populations (locations); C, clusters of sub-populations (groups).

**Figure 3:**
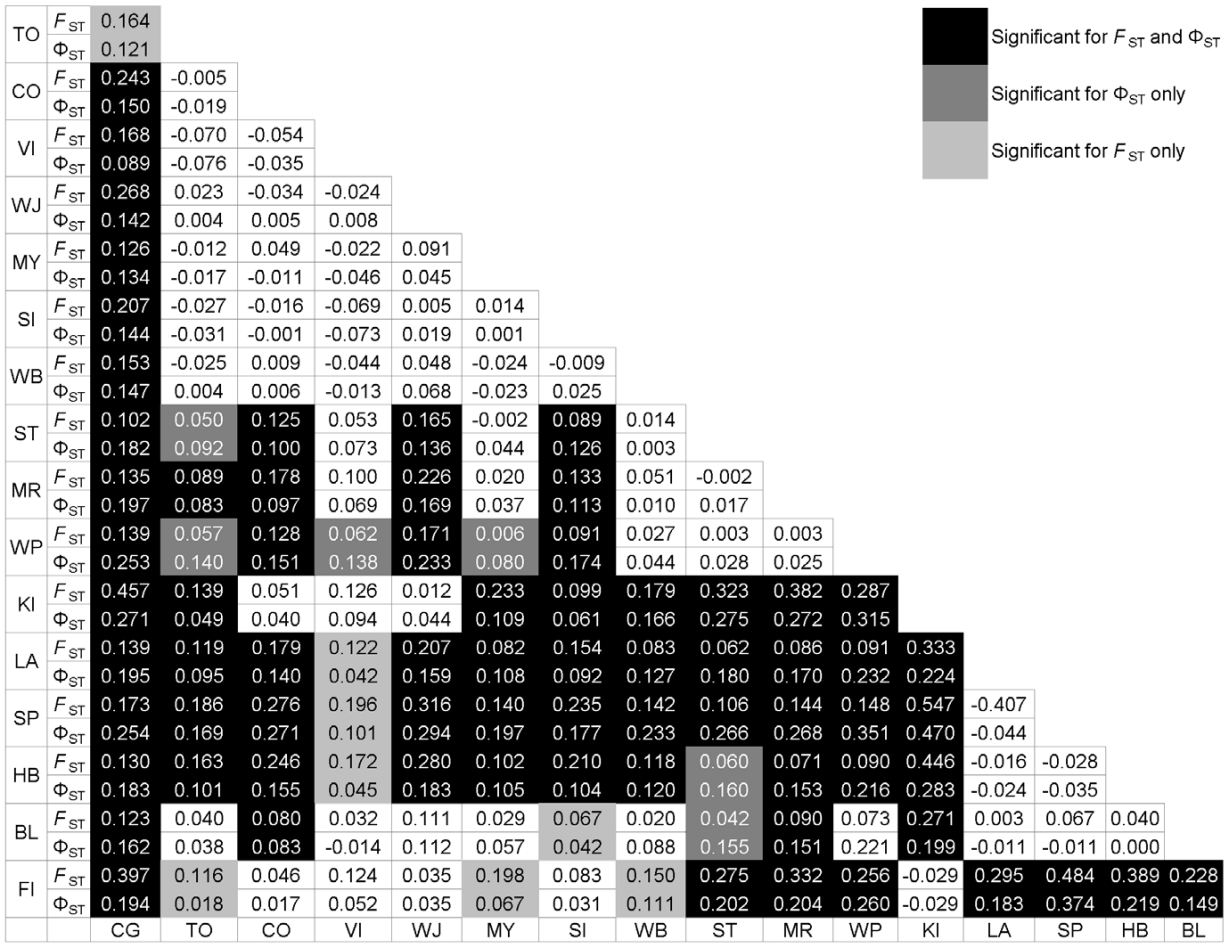
Pairwise *F*_ST_ and Φ_ST_ values between each pair of collection sites. Significant values are indicated by shading (P<0.05). Negative values are equivalent to zero, indicating no differentiation between samples.

Pair-wise comparisons were undertaken to determine specifically which collection sites were responsible for the variation detected among sites (Figure 3). All Flinders Island (FI) specimens were of the common H1 haplotype, as were all Kangaroo Island (KI) specimens except one that had H11 haplotype, otherwise found in one individual in each of three other sampling sites (MY, ST, WP). This low diversity is not unexpected for island populations [40], but renders unclear the relationships to the mainland and Tasmanian locations. Carnarvon Gorge (CG) was found to be significantly different from all other sites based on *F*_ST_ and all except the nearest site, Toowoomba (TO), based on Φ_ST_. This indicates ND5 evolutionary-timescale matrilineal differentiation of CG from the rest of the range except for Toowoomba. No significant differentiation was found among any of sites from mainland South Australia, but most were differentiated from most other mainland sites on both *F*_ST_ and Φ_ST_. Only Wright Bay (WB), which geographically links the South Australian sites to the more easterly mainland sites, showed little differentiation from either. Tasmanian sites were significantly differentiated from all mainland sites based on *F*_ST_ and Φ_ST_, but not from each other. Thus Carnarvon Gorge and Tasmania appear to be significantly isolated from the rest of the distribution, while South Australia appears to remain connected via limited gene flow potentially through the environs of Wright Bay. The isolation of Carnarvon Gorge and Tasmania from the rest of the mainland was examined more closely to estimate the time since the populations split and whether there is any remaining migration.

#### The population split between mainland Australia and Tasmania

The interpretation of IMa analysis (Table 5, Figures S2, S3, S4) has been described in detail previously [36] and is summarised in Materials and Methods. Not all six parameters of the full isolation-with-migration model could be reliably estimated using COI due to insufficient data: marginal distributions for some parameters (e.g. θ_2_, *m*2) did not converge over four runs and for others (*t*) did not reach zero (Table 5, Figure S2). The distinct peak of the marginal distribution of time since population divergence between mainland and Tasmania, *t*, was consistent over the runs, corresponding to 26–32 thousand years ago (Kya). However, four nested models that were not rejected by LRT for COI data (Table 6) suggested the time of the split around 53–124 Kya. ND5 data provided more resolution and yielded a more recent estimate of population split: 21 Kya (12–36 Kya 90% highest posterior density, HPD; Table 5 and Figure S3); all nested models were rejected. These results suggest that the split between mainland and Tasmanian mtDNA lineages occurs in the Late Pleistocene but somewhat predates the conventional date of Bass Strait formation [10 Kya, 41].

**Table 5:** Results of three IMa analyses.

Model		θ_1_	θ_2_	θ_A_	N_e(1)_, number of individuals	N_e(2)_, number of individuals	N_e(A)_, number of individuals	*m*_1 (2 to 1)_	*m*_2 (1 to 2)_	*t*	t, years
COI, Mainland (1) vs. Tasmania (2)	Multidimensional peak location	12.125	n/e	0.006	605,887	n/e	300	0.585	n/e	0.535	82,258
	Marginal peak location	7.762	n/e	0.010	387,918	n/e	483	2.288	n/e	0.194	29870
	Lower 90% HPD	2.297	n/e	0.010	114,804	n/e	483	0.016*	n/e	0.037*	5651*
	Upper 90% HPD	28.026	n/e	13.745	1,400,612	n/e	686,891	24.016*	n/e	3.498*	537,923*
ND5, Mainland (1) vs. Tasmania (2)	Multidimensional peak location	35.630	29.134	3.009	1,224,247	1,001,045	103,389	0.0001	0.0003	0.191	20,193
	Marginal peak location	34.087	19.748	2.983	1,171,238	678,545	102,504	0.002	1.677	0.198	20,880
	Lower 90% HPD	17.068	8.069*	0.910	586,459	277,266*	31,255	0.002	0.004*	0.116	12211
	Upper 90% HPD	67.245	49.444*	8.930	2,310,549	1,698,884*	306,841	1.734	4.960*	0.341	35,999
ND5, Mainland (1) vs. Carnarvon Gorge (2)	Multidimensional peak location	83.168	0.659	0.971	2,857,653	22,643	33,364	0.0001	n/e	0.198	20,880
	Marginal peak location	50.775	1.310	2.611	1,744,622	45,009	89,711	0.005	n/e	0.171	18,026
	Lower 90% HPD	31.313*	0.205	0.383	1,075,922*	7,042	13,166	0.005	n/e	0.057	5,973
	Upper 90% HPD	81.963*	7.120	9.954	2,816,258*	244,639	342,005	5.965	n/e	0.408	43,082

Coalescent estimates of relative effective sizes for contemporary populations (1), (2) and their ancestral population (A), relative immigration rates *m*1 into population (1), and *m*2 into (2), and relative time of split *t* between (1) and (2) are derived from combined parameter distributions of four IMa runs. Absolute time (t) is calculated assuming μ_COI_ of 6.504×10^−6^ or μ_ND5_ of 9.459×10^−6^ substitutions per year per locus, demographic estimates of N_e_ are calculated assuming generation time of 1 year; n/e indicates samples not estimated due to insufficient data (marginal distributions did not converge).

* HPD estimates are unreliable because the tail of the distribution did not reach zero.

**Table 6:** Tests and parameters estimated in IMa for nested models to identify the simplest model that explains the data.

Model	Log(P)	2LLR	df	P-value	*t*	t, years	θ_1_	θ_2_	θ_A_	*m*_1 (2 to 1)_	*m*_2 (1 to 2)_
θ_1_ θ_2_ Θ_A_ *m*_1_ *m*_2_	−5.64	-	-	-	0.535	82,259	12.1	19.3	0.006	0.585	4.499
θ_1_ θ_2_ θ_A_ *m*_1_ = *m*_2_	−5.99	0.701	1	0.4	0.806	123,925	10.1	17.1	0.005	1.088	1.088
θ_1_ θ_2_ θ_A_ *m*_1_ *m*_2_ = 0	−5.72	0.165	1§	0.68	0.344	52,892	13.3	12.2	0.01	3.916	0
θ_1_ = θ_2_ θ_A_ *m*_1_ *m*_2_	−5.85	0.417	1	0.52	0.345	53,045	13.0	13.0	0.01	3.9	0
θ_1_ = θ_2_ θ_A_ *m*_1_ = *m*_2_	−7.30	3.318	2	0.19	0.538	82,720	12.5	12.5	0.007	1.447	1.447

Models that were not rejected by LRT (P>0.05) are shown and all are for COI data: Mainland (1) versus Tasmania (2). ‘Log(P)’ is the posterior probability of the model given data, ‘2LLR’ = 2×(Log(P)_nested model_−Log(P)_full model_), ‘df’ is the difference in number of parameters between nested and full model except where marked with § (in which case models have distributions of 2LLR that are a mixture, see [36]), ‘P-value’ is the probability of achieving the test statistic (2LLR) by chance under the null model. Nested models were rejected for all ND5 analyses.

Migration between mainland and Tasmania is estimated to be extremely low (upper HPD<0.2 migrations per 1000 generations per gene copy for both COI and ND5; Table 5). Only ND5 data yielded reasonable estimates of both migration parameters, and these indicated that migration from Tasmania into mainland has been much less common than from mainland into Tasmania (immigration into Tasmania, *m*2>immigration into mainland, *m*1; Table 5).

#### The population split between Carnarvon Gorge and the remaining mainland sites

Analysis of mainland and Carnarvon Gorge populations using ND5 suggests there is a lack of gene flow from Carnarvon Gorge to the other mainland sites, but migration in the opposite direction could not be estimated (parameter distributions of replicate runs did not converge). Carnarvon Gorge is estimated to have split from the rest of the mainland about 18–21 Kya (6–43 Kya, 90% HPD; Table 5 and Figure S4). Thus, although this estimate is slightly later than the Tasmania-mainland split, we cannot reject simultaneous splitting of Tasmania and Carnarvon Gorge from the mainland population.

## Discussion

This study used mitochondrial loci COI and ND5 to investigate the population genetic structure and phylogeography of *H. merope* over the continent-wide range of the species. Using this genetic information, the relationship between the three recognised *H. merope* subspecies and the timing of splits between distinct populations in eastern Australia have been estimated. This knowledge aids in the interpretation of morphological and allozymic variation observed previously in *H. merope* [6], [7] as well as providing valuable baseline data for studies into climate change responses in butterflies.

### ND5 provides more resolution than does COI in *H. merope*

ND5 allowed a more resolving study of population genetic structure and phylogeography of *H. merope*, having approximately three times the sequence variation per nucleotide position than COI. It has been suggested that a generally faster evolutionary rate in invertebrate ND genes could be due to positive selection [42], [43]. However, there is also strong evidence for greatly elevated rates of mutation in the minor strand of mtDNA, where ND5 is located in most insects, than in the major strand where COI is found [44], [see also mechanistic studies 45], [46]. In the present ND5 data set, there were very few non-synonymous substitutions and no significant effects were detected by MacDonald-Kreitman tests, so the greater variation in ND5 than COI is consistent with elevated mutation rate rather than selection. Our data thus agree with previous suggestions that ND5 is relatively diverse at low taxonomic levels [17], [18]. However, our literature searches indicate that this locus has been used in relatively few intraspecific phylogeographies. This study illustrates the value of comparing several loci to identify those most resolving for particular studies, and ND5 provides a good candidate for comparison to COI.

### The population genetic structure of *H. merope*

The mtDNA throughout much of the range of the eastern mainland subspecies *H. m. merope* is essentially undifferentiated. However, there are two notable exceptions. Locations in the State of South Australia differ in haplotype frequencies from locations in the other eastern states, but appear to remain connected via Wright Bay. The geographically outlying northern site Carnarvon Gorge is similarly differentiated from all other locations, and appears to have split from the main population during the Late Pleistocene. There is no current migration from Carnarvon Gorge to the other sites. Although it was not possible to calculate a migration rate in the other direction, weak prevailing winds are unlikely to support migration across the 150–200 km of inhospitable habitat [6] and the differentiation in haplotype frequencies indicates this would be extremely low or non-existent.

The Tasmanian subspecies *H. m. salazar* has strong frequency differences from the mainland. Unfavourable habitat appears to have disrupted gene flow between the mainland and Tasmania during the Late Pleistocene but somewhat earlier than the conventional date of the formation of Bass Strait. Supporting this, an overview of the relevant palynological literature suggests that woodland and forest habitat between current mainland and Tasmania could have been severed considerably earlier than the inundation of Bass Strait (as much as 40 Kya) from the perspective of Lepidoptera dependent on woody vegetation [47]. However, the lower end of the range of population splitting times (Table 5) is close to the estimated date of the Bass Strait formation, so we cannot exclude the closing of the land bridge between the mainland and Tasmania as a relevant vicariance event. There is currently virtually no *H. merope* migration between Tasmania and the mainland, and any migration that may occur is far more likely to be from mainland to Tasmania than *vice versa*. This is consistent with the suggestion that occasional strong northerly winds could support limited one-way gene flow into Tasmania from the mainland population [6].

The Western Australian subspecies, *H. m. duboulayi*, is virtually as divergent in mtDNA from eastern individuals as is congener *H. penelope*. A similar conclusion is suggested by morphological and allozymic variation [6]. Attempts to interbreed *H. m. duboulayi* and *H. m. merope* have showed considerable reproductive isolation between the subspecies through hybrid zygote inviability and sterility [6]. All approaches therefore indicate that *H. m. duboulayi* is a distinct evolutionary unit from the other subspecies. This background establishes *H. m. duboulayi* as an important point of comparison with *H. m. merope* in terms of climate change biology.

### Reinterpreting morphological clines with knowledge of population genetic structure

The distinct lack of genetic structuring throughout most of the distribution along the east coast of mainland Australia indicates a level of gene flow that is consistent with reports of high dispersal potential and continuity of *H. merope* habitat [6] except where barriers to dispersal affect South Australia and Carnarvon Gorge. Similarly, minimal structure was observed in allozyme variation throughout most of this region [6]. The north-south clinal variation in morphological characters observed across this range [7] is therefore likely to reflect differential selection rather than be an artefact resulting from isolation-by-distance and genetic drift. This is supported further by the fact that Carnarvon Gorge (represented by the nearby Expedition Range in the 1970s sampling) appears to be an extension of the north-south cline in morphological characters despite a high level of differentiation at mitochondrial and allozyme loci resulting from a population split we estimate to have occurred during the Late Pleistocene.

In contrast, a selection hypothesis for the east-west cline is not obviously supported. Although the allozyme data detect differentiation of only Wilpena Pound from the rest of the contiguous distribution [6], the mitochondrial data reveal restricted gene flow between the entire South Australian region and the remainder of the range. This study therefore supports an alternative explanation for the east-west cline whereby historical glaciation is likely to have separated the South Australian region from the remainder of the range, allowing genetic drift to generate differences between the regions [7]. The cline may then have formed following subsequent reconnection of populations via the arid region surrounding Wright Bay because gene flow has been limited and some differentiation persists. It remains possible that the east-west cline is related to climatic variation such as yearly rainfall, but other studies are required to confirm this because the population genetic structure indicates that the alternative hypothesis is equally valid.

The interpretation of clinal variation using knowledge of mitochondrial phylogeography suggests that it would be valuable to revisit the study of morphological characters in *H. merope*, particularly in relation to those characters contributing to the north-south cline associated with winter humidity. As Pearse and Murray's collection sites were specifically located near weather stations [7], it is possible to seek shifts in the clinal distribution that may have occurred over 30 years of climate change.

### Evidence for potential selective pressure on allozymes PGI and PGM in *H. merope*

The *H. merope* population genetic structure was examined in the late 1970s using PGI, PGM and a third allozyme, aspartate aminotransferase [AAT, a.k.a. GOT, 6]. A similar picture to that derived from mtDNA was seen for the population splits, but significant allozyme differentiation of the northernmost South Australian location, Wilpena Pound (WP), is at odds with the considerable gene flow throughout the region apparent from mtDNA. Interestingly, the differentiation is driven strongly by PGI and PGM (differing significantly from 90% and 100% of other *H. m. merope* samples, respectively; contingency χ^2^, P<0.05), rather than AAT (differing from only 25%, P<0.05).

Wilpena Pound is geographically isolated by a floodplain that constitutes approximately 100 km of arid, inhospitable habitat [6] and, like Carnarvon Gorge, borders the desert regions of Australia and experiences highly variable rainfall (Australian Government Bureau of Meteorology, Index of Variability). This suggests that admixture with the rest of South Australia may be facilitated by intermittent flooding and decades of partial isolation between floods could potentially support genetic drift as well as selection. However, genetic drift seems an insufficient explanation as the differentiation at Wilpena Pound is reflected in neither mtDNA nor AAT allele frequencies and is higher than expected relative to Tasmania, despite long-term isolation of the latter (Tasmanian sites differed significantly from 10–71% of mainland sites, with no pattern among allozymes or sites; P<0.05). Wilpena Pound is located at the extreme of the habitable mainland range. Due to the hooked shape of the distribution, this is inland approximately halfway along the latitudinal gradient occupied by the species (Figure 1). This is well outside the range of any other *Heteronympha* species [8] suggesting partial isolation could facilitate a greater rate of adaptation than in coastal sites of similar latitude which experience higher gene flow and potential selective swamping [reviewed by 48].

Consistent with the suggestion of selective pressure in *H. merope*, variation in PGI and PGM has been associated with variation in flight ability and fitness measures across different thermal environments in many insects including butterflies [49]–[53], but no such associations have been found for AAT. Further studies of PGI and PGM function in *H. merope* may therefore reveal a relationship between allozyme genotype, climatic variables and physiological performance in this species. Such studies could involve the comparison of flight ability, thermal tolerance and PGI and PGM genotypes among specimens from Wilpena Pound, Carnarvon Gorge, the contiguous population, Tasmania, and Western Australia. A selection hypothesis could also be further examined by detailed population genetic studies that compare PGI and PGM to a greater number of reference loci that are likely to be selectively neutral.

### *H. merope* as a model for climate change research

The response of an organism to environmental change depends on how such change interacts with species characteristics including thermal biology and dispersal ability [54], [55]. In order to predict the potential impacts of forces such as climate change and habitat fragmentation, it is therefore necessary to understand adaptive potential and dispersal ability for species of interest [56], [57]. Knowledge of historical changes in range distribution supports this understanding in several key ways [58]–[62]. First, insight into past responses can be gained through examining historical changes in distribution in the context of climate records and changes in habitat structure. Second, experimentally-derived mechanistic models can be tested by modelling the effects of past environmental change and comparing the outcomes to known changes in range distribution. Third, an understanding of spatial genetics and phylogeography derived from neutral genetic markers provides a backdrop for studies of adaptive loci, helping to distinguish between biased dispersal and *in situ* adaptation in the study of thermal biology and dispersal ability.

Understanding of the mitochondrial population genetic structure of *H. merope* has provided such context, paving the way for a powerful approach towards predictive modelling of future range distributions in butterflies. Having defined the level of isolation of Carnarvon Gorge, South Australia and Tasmania, and shown there is no apparent barrier to gene flow among the remaining sites, it has been possible to investigate whether patterns of variation in other characters are likely to reflect genetic drift or adaptive change. This approach has yielded evidence for the adaptive significance of previously studied wing characters and allozymes in *H. merope* as well as providing the required context for examining other traits in the future. Ranges along the east coast of Australia are typical of a vast number of butterfly species and in addition, the genus *Heteronympha* contains a number of species with altitudinally- or habitat-restricted distributions [8]. *Heteronympha merope* therefore provides an ideal model for Australian butterflies as well as for investigating adaptive traits in the contrasted settings of both latitudinal gradients and isolated populations. Such a model will be extremely valuable in the predicting the impacts of climate change and habitat fragmentation on butterflies and other species.

## Supporting Information

Figure S1 Median Joining Network derived from (A) COI and (B) combined COI and ND5 sequences. COI data is from 166 specimens and combined COI and ND5 data is from 151 specimens. Interpretation of these diagrams is described in the Figure 2 legend in the main manuscript.
(9.34 MB EPS)

Figure S2 Marginal posterior parameter distributions from IMa analysis for mainland (1) and Tasmania (2) populations based on COI. Data from the four replicates are combined. Parameters (described in Methods) are Φ 1 = mainland population size, Φ 2 = Tasmanian population size, Φ A = ancestral population size, m1 = rate of migration into mainland since split, m2 = rate of migration into Tasmania since split, t = time since population divergence.
(0.29 MB TIF)

Figure S3 Marginal posterior parameter distributions from IMa analysis for mainland (1) and Tasmania (2) populations based on ND5. Data from the four replicates are combined. Parameters are defined in the Figure S2 legend.
(0.31 MB TIF)

Figure S4 Marginal posterior parameter distributions from IMa analysis for mainland (1) and Carnarvon Gorge (2) populations based on ND5. Data from the five replicates are combined. Parameters m1 = rate of migration into mainland since split and m2 = rate of migration into Carnarvon Gorge since split. Other parameters are defined in the Figure S2 legend.
(0.30 MB TIF)

Table S1 *H. m. merope* and *H. m. salazar* COI haplotype sequences compared to the most common haplotype, H1. Site position is relative to the first site of *H. merope* complete COI sequence AY218243 available through the National Center for Biotechnology Information (www.ncbi.nlm.nih.gov). *Non-synonymous substitutions: G256A, Val85Met; G343A, Gly114Ser; A452G, Asn150Ser. Genbank accession numbers are provided in Table S3 for these and the *H. m. duboulayi* and *H. penelope* haplotypes.
(0.05 MB DOC)

Table S2 *H. m. merope* and *H. m. salazar* ND5 haplotype sequences compared to the most common haplotype, H1. Site position is relative to the first site of Lepidopteran ND5 sequences available through the National Center for Biotechnology Information (www.ncbi.nlm.nih.gov; e.g AB107980, EU037820). *Non-synonymous substitutions: A85G, Ile29Val; G170T, Ser57Met; T257C, Ile86Thr. Genbank accession numbers are provided in Table S3 for these and the *H. m. duboulayi* haplotypes, as well as for haplotypes of the other three species sequenced for this study.
(0.15 MB DOC)

Table S3 Haplotype summary and Genbank accession numbers for all *Heteronympha* specimens used in this study.
(0.08 MB XLS)
