# Supplementary figures and images for: Mitochondrial DNA Indicates Late Pleistocene Divergence of Populations of Heteronympha merope, an Emerging Model in Environmental Change Biology
Source: PLoS One. 2009 Nov 24;4(11):e7950. doi: 10.1371/journal.pone.0007950 (PMC2776993; doi:10.1371/journal.pone.0007950)

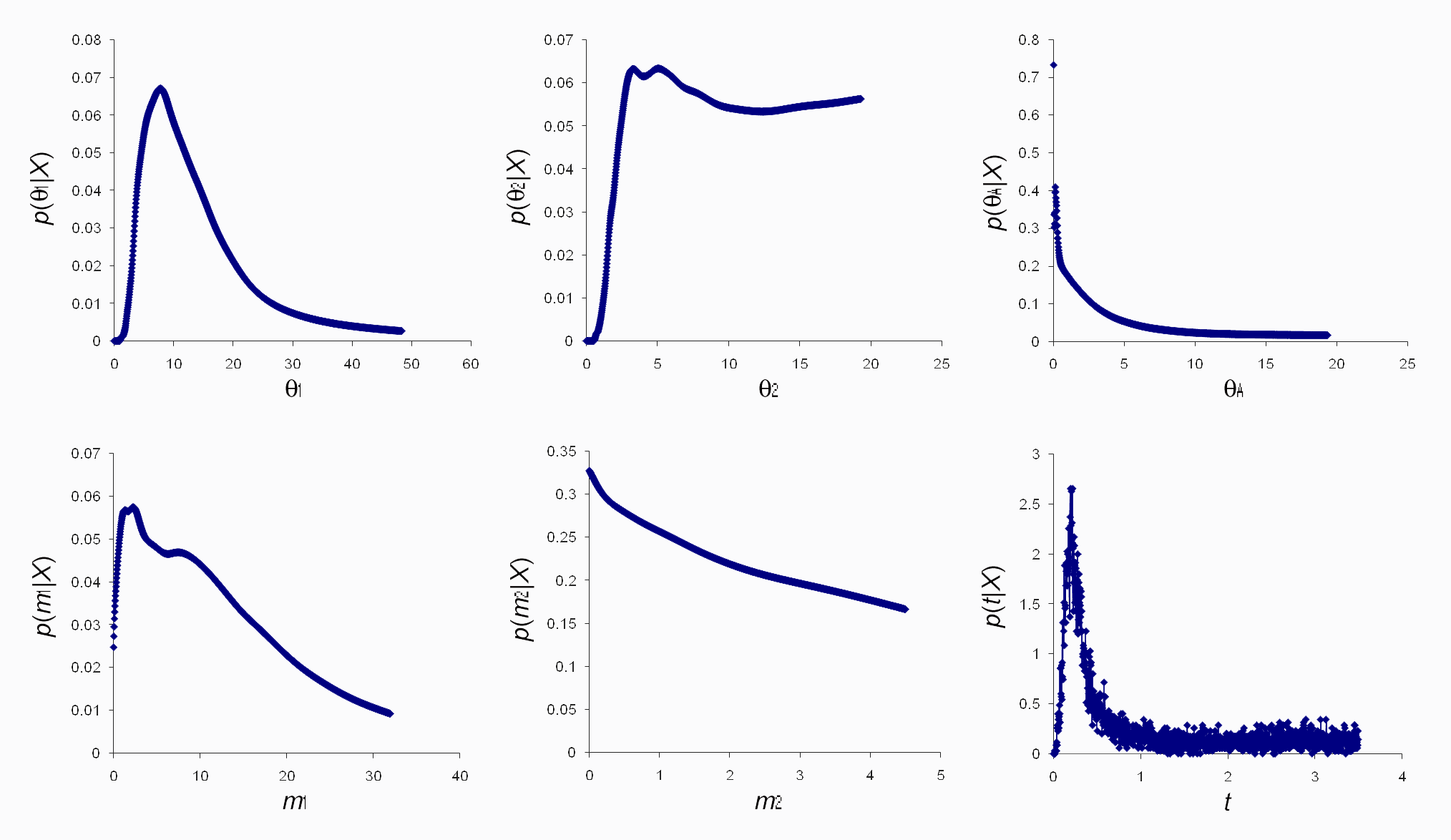

Supplement: Figure S2 — Marginal posterior parameter distributions from IMa analysis for mainland (1) and Tasmania (2) populations based on COI. Data from the four replicates are combined. Parameters (described in Methods) are Φ 1 = mainland population size, Φ 2 = Tasmanian population size, Φ A = ancestral population size, m1 = rate of migration into mainland since split, m2 = rate of migration into Tasmania since split, t = time since population divergence. (0.29 MB TIF) [file pone.0007950.s002.tif]

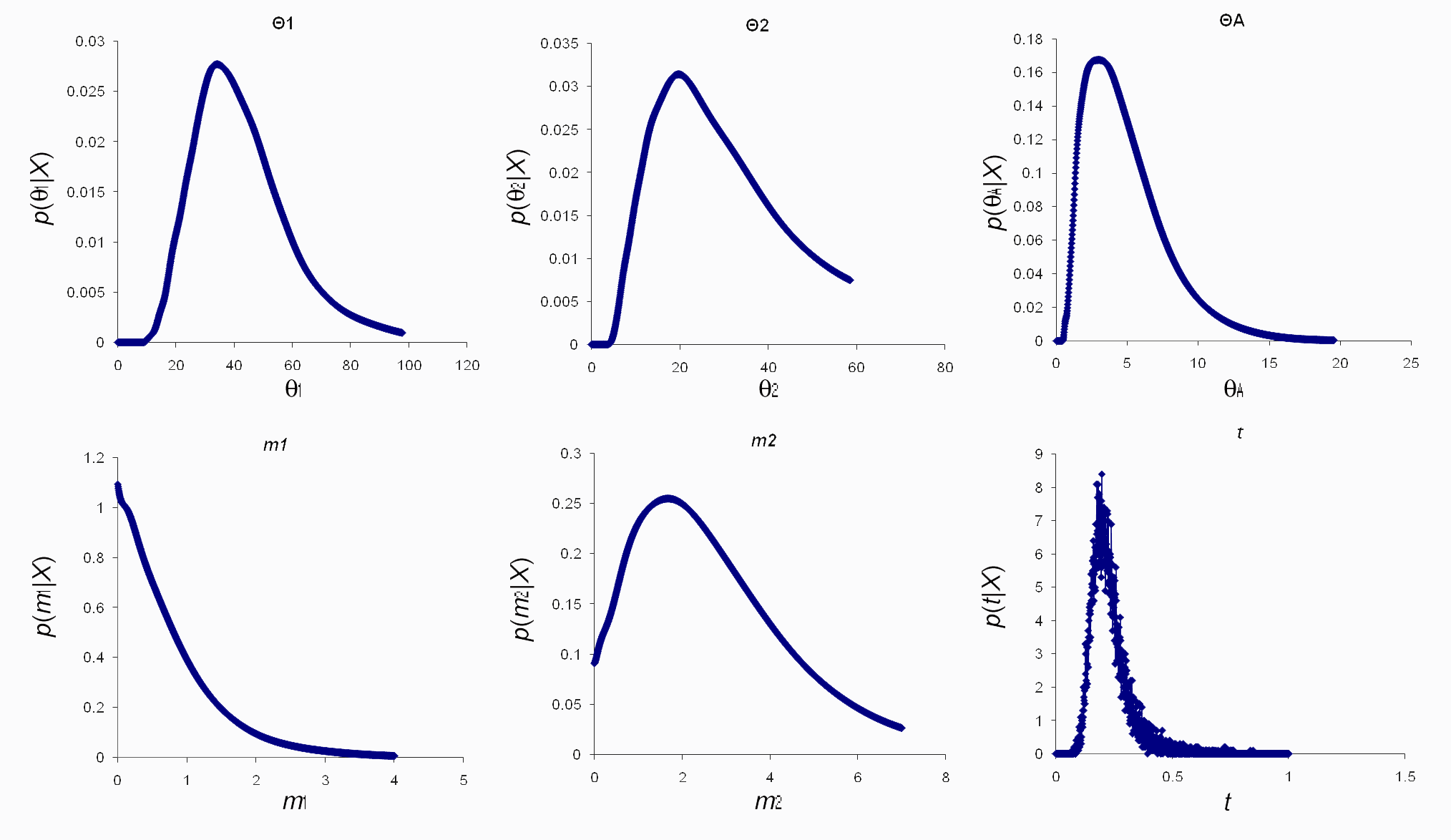

Supplement: Figure S3 — Marginal posterior parameter distributions from IMa analysis for mainland (1) and Tasmania (2) populations based on ND5. Data from the four replicates are combined. Parameters are defined in the Figure S2 legend. (0.31 MB TIF) [file pone.0007950.s003.tif]

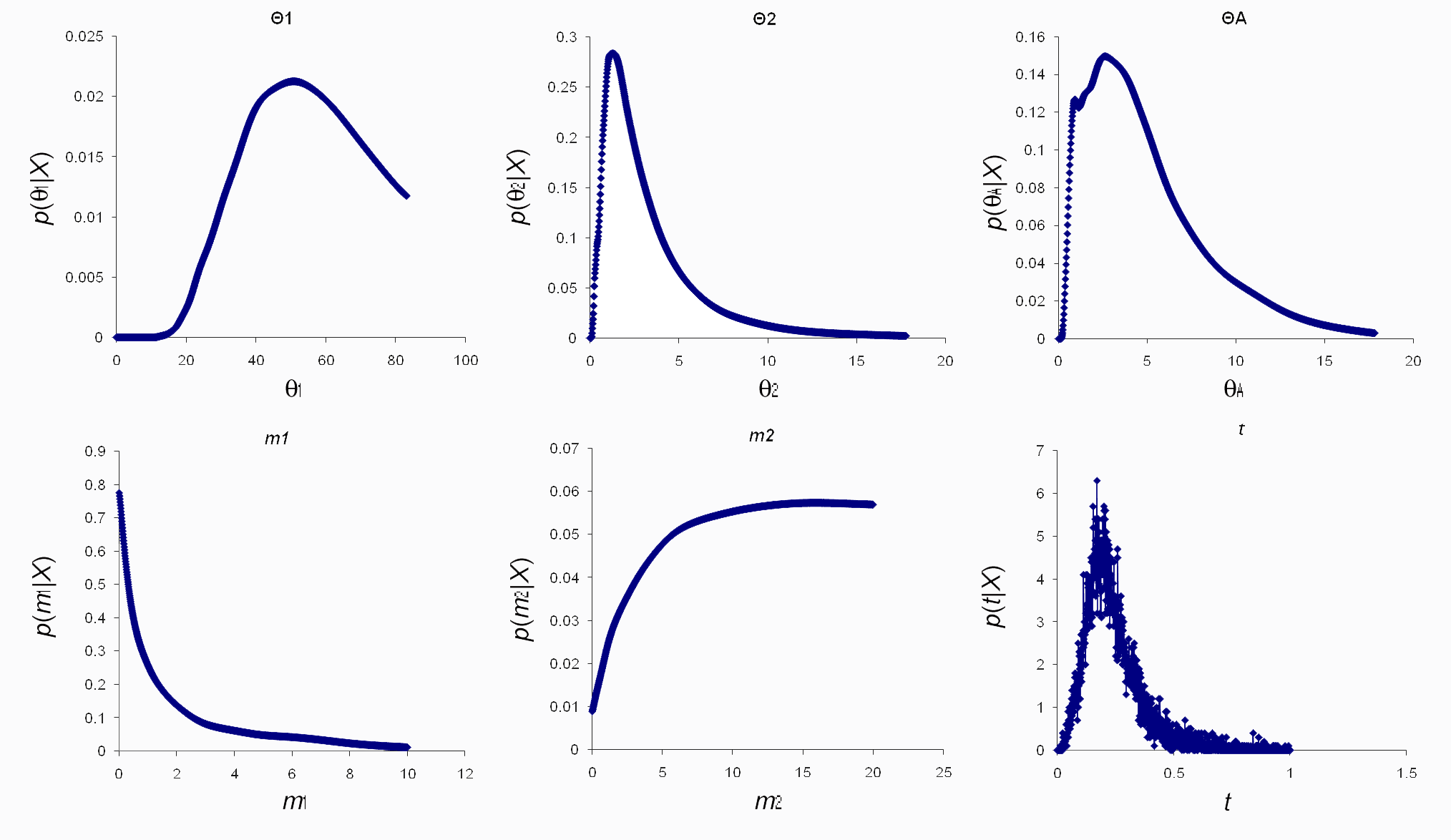

Supplement: Figure S4 — Marginal posterior parameter distributions from IMa analysis for mainland (1) and Carnarvon Gorge (2) populations based on ND5. Data from the five replicates are combined. Parameters m1 = rate of migration into mainland since split and m2 = rate of migration into Carnarvon Gorge since split. Other parameters are defined in the Figure S2 legend. (0.30 MB TIF) [file pone.0007950.s004.tif]
