# Supplementary material for: Mitochondrial DNA Indicates Late Pleistocene Divergence of Populations of Heteronympha merope, an Emerging Model in Environmental Change Biology
Source: PLoS One. 2009 Nov 24;4(11):e7950. doi: 10.1371/journal.pone.0007950 (PMC2776993; doi:10.1371/journal.pone.0007950)
Supplement: Table S1 — H. m. merope and H. m. salazar COI haplotype sequences compared to the most common haplotype, H1. Site position is relative to the first site of H. merope complete COI sequence AY218243 available through the National Center for Biotechnology Information (www.ncbi.nlm.nih.gov). *Non-synonymous substitutions: G256A, Val85Met; G343A, Gly114Ser; A452G, Asn150Ser. Genbank accession numbers are provided in Table S3 for these and the H. m. duboulayi and H. penelope haplotypes. (0.05 MB DOC) [file pone.0007950.s005.doc]

**Table S1. *H. m. merope* and *H. m. salazar*** COI haplotype sequences compared to the most common haplotype, H1.

|  | **246** | **256*** | **288** | **343*** | **378** | **438** | **452*** | **507** | **546** | **609** | **645** |
| --- | --- | --- | --- | --- | --- | --- | --- | --- | --- | --- | --- |
| **H1** | T | G | C | G | A | C | A | A | C | C | A |
| **H2** | . | . | . | . | . | . | . | . | . | . | G |
| **H3** | C | . | . | . | . | T | . | . | . | . | . |
| **H4** | . | . | . | . | . | . | . | . | . | T | . |
| **H5** | . | . | . | . | . | . | . | . | T | . | . |
| **H6** | . | A | . | . | . | . | . | . | . | . | . |
| **H7** | . | A | T | . | . | . | . | . | . | . | . |
| **H8** | . | . | . | . | . | . | G | . | . | . | . |
| **H9** | . | . | . | . | G | . | . | . | . | . | . |
| **H10** | . | . | T | . | . | . | . | . | . | . | . |
| **H11** | . | . | . | A | . | . | . | . | . | . | . |
| **H12** | . | . | . | . | . | . | . | G | . | . | . |
| **H13** | . | . | . | . | . | T | . | . | . | . | . |

Site position is relative to the first site of *H. merope* complete COI sequence AY218243 available through the National Center for Biotechnology Information (www.ncbi.nlm.nih.gov). *Non-synonymous substitutions: G256A, Val85Met; G343A, Gly114Ser; A452G, Asn150Ser. Genbank accession numbers are provided in Table S3 for these and the *H. m. duboulayi* and *H. penelope* haplotypes.
