# Supplementary material for: Mitochondrial DNA Indicates Late Pleistocene Divergence of Populations of Heteronympha merope, an Emerging Model in Environmental Change Biology
Source: PLoS One. 2009 Nov 24;4(11):e7950. doi: 10.1371/journal.pone.0007950 (PMC2776993; doi:10.1371/journal.pone.0007950)
Supplement: Table S2 — H. m. merope and H. m. salazar ND5 haplotype sequences compared to the most common haplotype, H1. Site position is relative to the first site of Lepidopteran ND5 sequences available through the National Center for Biotechnology Information (www.ncbi.nlm.nih.gov; e.g AB107980, EU037820). *Non-synonymous substitutions: A85G, Ile29Val; G170T, Ser57Met; T257C, Ile86Thr. Genbank accession numbers are provided in Table S3 for these and the H. m. duboulayi haplotypes, as well as for haplotypes of the other three species sequenced for this study. (0.15 MB DOC) [file pone.0007950.s006.doc]

**Table S2. *H. m. merope* and *H. m. salazar*** ND5 haplotype sequences compared to the most common haplotype, H1.

|  | **39** | **48** | **66** | **85*** | **87** | **108** | **132** | **150** | **170*** | **177** | **180** | **186** | **198** | **210** | **216** | **249** | **257*** | **270** | **279** | **312** | **339** | **342** | **366** | **369** | **373** |
| --- | --- | --- | --- | --- | --- | --- | --- | --- | --- | --- | --- | --- | --- | --- | --- | --- | --- | --- | --- | --- | --- | --- | --- | --- | --- |
| **H1** | T | C | A | A | T | T | T | G | G | T | T | A | T | T | G | T | T | C | A | T | T | T | C | T | T |
| **H2** | . | . | . | . | . | . | . | . | . | . | . | . | . | C | . | . | . | . | . | . | . | . | . | . | . |
| **H3** | . | T | . | . | . | . | . | . | . | . | . | . | . | . | . | . | . | . | . | . | . | . | . | . | . |
| **H4** | . | . | . | . | . | . | . | . | . | . | . | . | . | C | . | . | . | T | . | . | . | . | . | C | . |
| **H5** | . | . | G | . | . | . | . | . | . | . | . | . | . | . | . | . | . | . | . | . | . | . | . | . | . |
| **H6** | . | . | . | . | . | . | . | . | . | . | . | . | . | C | . | . | . | T | . | . | . | . | . | . | . |
| **H7** | . | . | . | . | . | . | . | . | . | . | . | G | . | C | . | . | . | T | . | . | . | . | . | . | . |
| **H8** | . | . | . | . | . | . | . | . | . | . | . | . | . | . | . | . | . | . | G | . | . | . | . | . | . |
| **H9** | . | . | . | . | . | . | . | . | . | . | . | . | . | . | T | . | . | . | . | . | . | . | . | . | . |
| **H10** | . | T | . | . | . | . | . | . | . | . | . | . | . | . | . | . | . | . | . | . | . | . | . | . | C |
| **H11** | . | . | . | . | . | . | . | . | . | . | . | . | . | . | . | . | . | T | . | . | . | . | . | . | . |
| **H12** | . | T | . | . | . | . | . | A | . | . | . | . | . | . | . | . | . | . | . | . | . | . | . | . | . |
| **H13** | . | . | . | . | . | . | . | . | . | . | . | . | . | . | . | . | . | . | . | . | . | . | T | . | . |
| **H14** | . | T | . | . | . | . | . | . | . | . | . | . | . | . | . | G | . | . | . | . | . | . | . | . | . |
| **H15** | . | . | . | . | . | . | C | . | . | . | . | . | . | . | . | . | . | . | . | . | . | . | . | . | . |
| **H16** | . | . | . | G | . | . | . | . | . | . | . | . | . | . | . | . | . | . | . | . | . | . | . | . | . |
| **H17** | . | . | . | . | . | . | . | . | . | . | . | . | C | C | . | . | . | T | . | . | . | . | . | . | . |
| **H18** | . | . | . | . | . | . | . | . | . | . | . | . | . | C | T | . | . | T | . | . | . | . | . | . | . |
| **H19** | . | . | . | . | . | C | . | . | . | . | . | . | . | . | . | . | . | . | . | . | . | . | . | . | . |
| **H20** | . | T | . | . | . | . | . | . | . | C | . | . | . | . | . | . | . | . | . | . | . | . | . | . | . |
| **H21** | . | T | . | . | . | . | . | . | . | . | . | . | . | . | . | . | . | . | . | . | . | . | T | . | . |
| **H22** | C | . | . | . | . | . | . | . | . | . | . | . | . | . | . | . | . | . | . | . | . | . | . | . | . |
| **H23** | . | . | . | . | . | . | . | . | . | . | C | . | . | . | . | . | . | . | . | . | . | . | . | . | . |
| **H24** | . | . | . | . | . | . | . | . | . | . | . | . | . | . | . | . | C | . | . | . | . | . | . | . | . |
| **H25** | . | T | . | . | . | . | . | . | . | . | . | . | . | . | . | . | C | . | . | . | . | . | . | . | . |
| **H26** | . | . | . | . | C | . | . | . | . | . | . | . | . | C | . | . | . | T | . | . | . | . | . | . | . |
| **H27** | . | T | . | . | . | . | . | . | T | . | . | . | . | . | . | . | . | . | . | . | . | . | . | . | . |
| **H28** | . | . | . | . | . | . | . | . | . | . | . | . | . | . | . | . | . | . | . | . | C | . | . | . | . |
| **H29** | . | . | . | . | . | . | . | . | . | . | . | . | . | . | . | . | . | . | . | . | . | C | T | . | . |
| **H30** | . | . | . | . | . | . | . | . | . | . | . | . | . | . | . | . | . | . | . | C | . | . | . | . | . |
| **H31** | . | T | . | . | . | . | . | . | . | . | . | . | . | . | . | . | . | T | . | . | . | . | . | . | . |

Site position is relative to the first site of Lepidopteran ND5 sequences available through the National Center for Biotechnology Information (www.ncbi.nlm.nih.gov; e.g AB107980, EU037820). *Non-synonymous substitutions: A85G, Ile29Val; G170T, Ser57Met; T257C, Ile86Thr. Genbank accession numbers are provided in Table S3 for these and the *H. m. duboulayi* haplotypes, as well as for haplotypes of the other three species sequenced for this study.
